# A Moving Source of Matrix Components Is Essential for *De Novo* Basement Membrane Formation

**DOI:** 10.1016/j.cub.2017.10.001

**Published:** 2017-11-20

**Authors:** Yutaka Matsubayashi, Adam Louani, Anca Dragu, Besaiz J. Sánchez-Sánchez, Eduardo Serna-Morales, Lawrence Yolland, Attila Gyoergy, Gema Vizcay, Roland A. Fleck, John M. Heddleston, Teng-Leong Chew, Daria E. Siekhaus, Brian M. Stramer

**Affiliations:** 1 Randall Centre for Cell and Molecular Biophysics, King’s College London, London SE1 1UL, UK; 2 Institute of Science and Technology, Am Campus 1, 3400 Klosterneuburg, Austria; 3 Centre for Ultrastructure Imaging, King’s College London, London SE1 1UL, UK; 4 Advanced Imaging Center, Howard Hughes Medical Institute, Janelia Research Campus, Ashburn, VA 20147, USA

**Keywords:** basement membrane, extracellular matrix, macrophage, hemocyte, cell migration, *Drosophila*, morphogenesis, collagen IV, laminin, perlecan

## Abstract

The basement membrane (BM) is a thin layer of extracellular matrix (ECM) beneath nearly all epithelial cell types that is critical for cellular and tissue function. It is composed of numerous components conserved among all bilaterians [1]; however, it is unknown how all of these components are generated and subsequently constructed to form a fully mature BM in the living animal. Although BM formation is thought to simply involve a process of self-assembly [2], this concept suffers from a number of logistical issues when considering its construction *in vivo*. First, incorporation of BM components appears to be hierarchical [3, 4, 5], yet it is unclear whether their production during embryogenesis must also be regulated in a temporal fashion. Second, many BM proteins are produced not only by the cells residing on the BM but also by surrounding cell types [6, 7, 8, 9], and it is unclear how large, possibly insoluble protein complexes [10] are delivered into the matrix. Here we exploit our ability to live image and genetically dissect *de novo* BM formation during *Drosophila* development. This reveals that there is a temporal hierarchy of BM protein production that is essential for proper component incorporation. Furthermore, we show that BM components require secretion by migrating macrophages (hemocytes) during their developmental dispersal, which is critical for embryogenesis. Indeed, hemocyte migration is essential to deliver a subset of ECM components evenly throughout the embryo. This reveals that *de novo* BM construction requires a combination of both production and distribution logistics allowing for the timely delivery of core components.

## Highlights

• Macrophages are major producers of basement membrane in the *Drosophila* embryo
• Basement membrane components require hierarchical deposition during development
• Macrophage migration is essential to evenly deliver a subset of matrix components
• Uneven macrophage dispersal leads to uneven matrix incorporation and lethality

## Teaser

*Drosophila* macrophages undergo stereotyped developmental migrations to evenly populate the embryo. Matsubayashi et al. reveal that macrophages are the biggest producers of extracellular matrix in the embryo and that their dispersal is essential to evenly distribute a subset of matrix components during development.

## Results and Discussion

To analyze *de novo* basement membrane (BM) formation, we exploited developing *Drosophila* embryos. We first analyzed the developmental profile of BM components from the *Drosophila* model organism Encyclopedia of DNA Elements (modENCODE) project [11]. This revealed that, while Laminin mRNAs are observed early in development, extracellular matrix (ECM) components associated with a mature BM, such as Collagen IV (Vkg in *Drosophila*) and Perlecan (Trol in *Drosophila*), are expressed later (Figure S1A), suggesting that there is a temporal hierarchy of BM production during embryogenesis.

**Figure 1:**
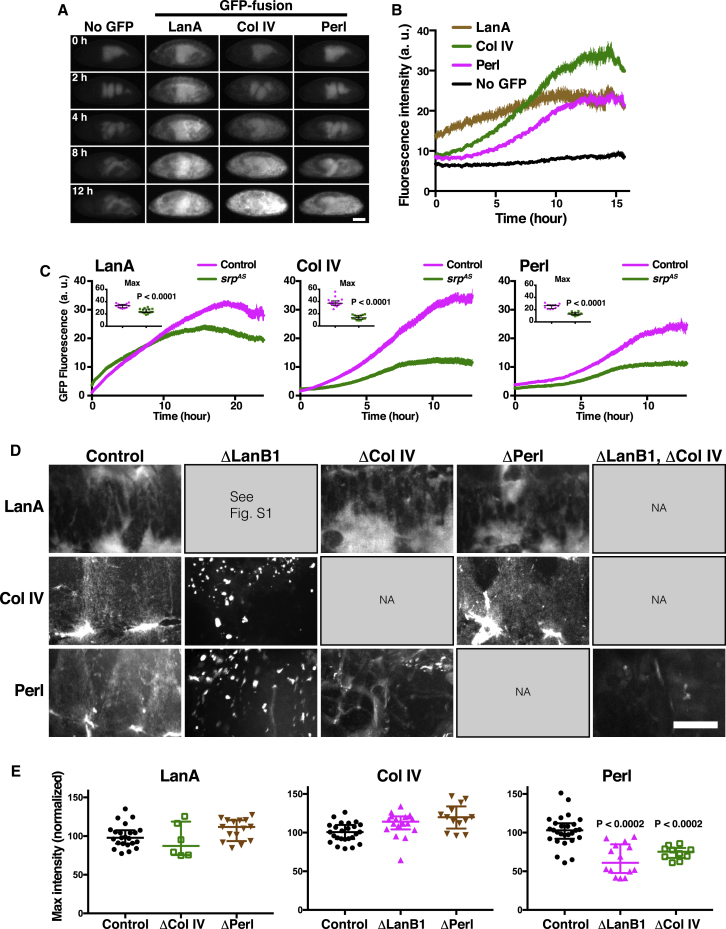
Temporal Hierarchy of BM Component Expression during Embryogenesis (A) Time-lapse imaging of *Drosophila* embryos expressing GFP-tagged BM components Laminin α (LanA), Collagen IV (Col IV), and Perlecan (Perl). Scale bar, 100 μm. (B) Quantification of embryonic fluorescence of BM components. Note that LanA is expressed first, followed by Col IV, and finally Perl. Mean ± SEM. (C) Quantification of embryonic fluorescence of BM components in control and *srp*^*AS*^ embryos. Mean ± SEM. Insets show quantification of the maximum fluorescence in each single embryo. Bars in the insets indicate median ± interquartile range. (D) Localization of GFP-tagged BM proteins on the ventral surface of the nerve cord in mutant backgrounds of opposite components. LanA-GFP and Col IV-GFP were imaged at stage 15, while Perl-GFP was imaged at stage 17 of development. Scale bar, 20 μm. (E) Quantification of embryonic fluorescence of GFP-tagged BM components in mutant backgrounds. Bars indicate median ± interquartile range. See also Figure S1, Table S1, and Movie S1.

We subsequently examined embryonic BM protein production using endogenously tagged BM fly lines. We used homozygous viable GFP-protein traps in Collagen IV (Col IV) [12] and Perlecan (Perl) [13] as well as a recently generated line containing GFP-tagged Lamininα (LanA) [14]. This LanA-GFP is capable of biochemically interacting with other Laminin subunits to form a mature Laminin trimer [14], and it rescued LanA mutant embryos (Figure S1B). Furthermore, when expressed in a Lamininβ (LanB1) mutant background, LanA levels were severely reduced (Figures S1C and S1D), suggesting that subunit trimerization is indeed essential for Laminin production and secretion [15]. Using these GFP-tagged lines, we analyzed the dynamics of BM production by quantifying GFP intensity over time during development (Movie S1). This revealed that expression of BM components peaked immediately prior to embryonic hatching. Furthermore, components showed precise temporal regulation with LanA expressed first, followed by Col IV, and finally Perl (Figures 1A and 1B). We also examined a second GFP-tagged construct of the sole *Drosophila* Lamininβ isoform (LanB1), which was previously confirmed to be fully functional [14], and this also revealed Laminin expression to occur prior to Col IV or Perl (Figure S1E).

In *Drosophila* embryos, hemocytes are known to produce BM [16, 17, 18]. However, it has been unclear what proportion of the embryonic BM is hemocyte dependent. When we expressed the GFP-tagged BM proteins in a mutant background in which hemocytes failed to develop [19], we revealed that BM components are differentially hemocyte dependent. This showed that 70% of Col IV and 50% of Perl are dependent on hemocytes. In contrast, hemocytes contribute only 30% of embryonic LanA, with most of the hemocyte-derived Laminin induced at later stages of development (Figures 1C and S1F). As the mesoderm expresses LanA [17], we hypothesize that its early expression is likely dependent on this tissue. For Col IV and Perl, the remaining protein was expressed in the fat body at late stages of development (Figure S1F), which is known to be the major source of larval BM [9].

To investigate the functional importance of the temporal hierarchy of BM component expression, we generated embryos expressing the GFP-tagged LanA, Col IV, and Perl in all possible mutant backgrounds of opposite components. This revealed that, while LanA incorporation or levels were unaffected by the absence of subsequent components (Figures 1D and 1E), Col IV and Perl formed disorganized extracellular deposits in the absence of Laminin (Figures 1D, S1G, and S1H). We hypothesize that these aggregates are the specific result of Col IV aggregation, as the Perl deposits were absent in a Col IV/Laminin double mutant (Figure 1D). Finally, Perl, which is expressed last in the temporal hierarchy, required prior production of Laminin and Col IV for proper expression and incorporation into the BM (Figures 1D and 1E), which is similar to what was previously reported [20]. These results suggest that proper *de novo* BM formation requires temporal regulation of component production. A similar temporal hierarchy of BM production may be critical for BM formation in other species, as disorganized ECM deposits have also been observed in *laminin* mutant mice [21] and *C. elegans* [22].

**Figure 2:**
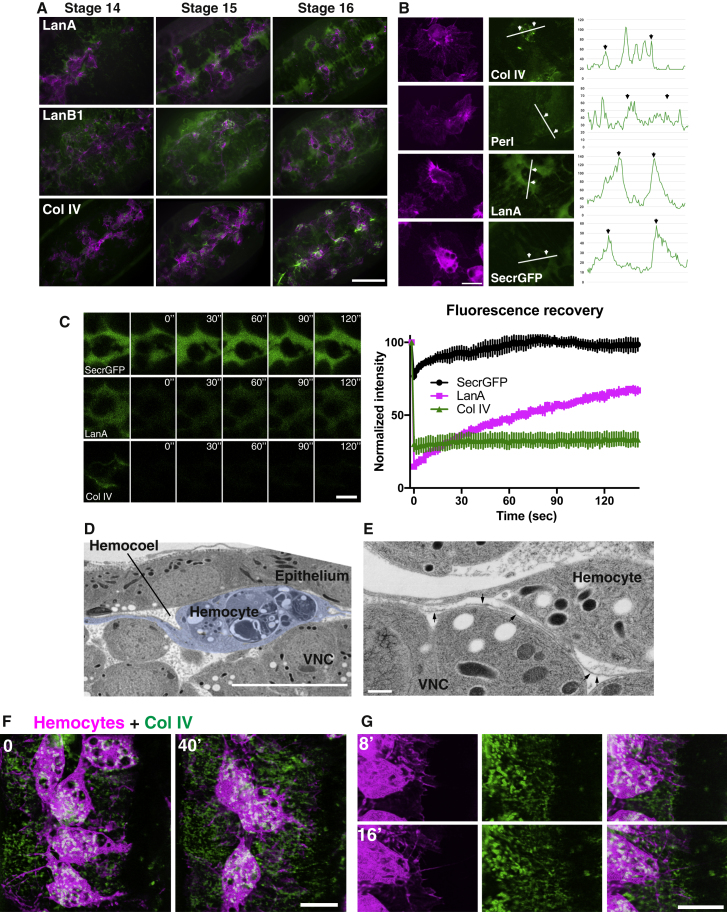
During BM Deposition, LanA Is Diffusing in the Embryonic Hemocoel while Col IV and Perl Are Locally Deposited (A) Confocal imaging of GFP-tagged BM proteins (green) and hemocytes (magenta) during development. Scale bar, 50 μm. (B) High-magnification images of LanA-GFP (stage 15), secreted-GFP (SecrGFP) (stage 15), Col IV-GFP (stage 16), and Perl-GFP (stage 17). Scale bar, 10 μm. Graphs are line scans of BM component fluorescence with arrows highlighting the border of the hemocyte cell body. (C) FRAP analysis of secreted-GFP, LanA-GFP, and Col IV-GFP in stage 15 embryos at the midline of the VNC. Mean ± SEM. Scale bar, 10 μm. (D) TEM of the ventral hemocoel of a stage 15 embryo, with the hemocyte highlighted in blue. Note the hemocyte squeezing between the epithelium and VNC within the narrow hemocoel. Scale bar, 10 μm. (E) TEM of a stage 16 hemocyte migrating along the VNC reveals BM material (arrows) beneath migrating cells. Scale bar, 2 μm. (F and G) Time-lapse imaging by lattice light-sheet microscopy of Col IV deposition (green) and hemocytes (magenta) at stage 14 of embryogenesis, as hemocytes migrate laterally from the ventral midline. (F) Low magnification. (G) High-magnification view highlighting the correlation between the leading edges of hemocytes and Col IV deposition. Scale bars, 10 μm. See also Figure S2, Table S1, and Movies S2 and S3.

We also observed differences in the appearance of Col IV and Laminin in the wild-type background, with Laminin showing a much more diffuse distribution (Figures 1D and 2A). We therefore investigated these differences by time-lapse microscopy during hemocyte migration along the ventral nerve cord (VNC), which is a known migratory route that is readily amenable to live imaging [23, 24, 25]. Both LanA and LanB1 subunits were observed to form “halos” of graded expression surrounding migrating hemocytes, with trails of Laminin forming as cells moved within the acellular fluid-filled cavity of the embryo (hemocoel) (Figures 2A and 2B; Movie S2, part 1). These halos of Laminin were identical to expression of secreted-GFP, suggesting that Laminin is simply filling the hemocoel (Figure 2B). In contrast, while Col IV and Perl decorated the surface of the VNC, there was no observable fluorescence filling the hemocoel (Figures 2A and 2B; Movie S2, part 2). We subsequently examined whether the differences in BM component localization were the result of their differing diffusive characteristics by performing fluorescence recovery after photobleaching (FRAP) analysis. This showed that LanA had a significant mobile fraction unlike Col IV, which failed to show any recovery (Figure 2C). To understand why Laminin formed halos surrounding hemocytes along the VNC, we examined the ventral hemocoel by transmission electron microscopy (TEM). This revealed that the ventral hemocoel is highly confined, with the VNC in physical contact with the overlying epithelium (Figure 2D). Therefore the halos of Laminin and its trails following hemocyte movement represent hemocytes separating the VNC from the overlying epithelium, allowing Laminin diffusion. These data highlight that different BM components have distinct diffusive properties within the developing embryo.

The apparent absence of soluble Col IV in the hemocoel suggested that Col IV might require a local mechanism of deposition by hemocytes. However, while it was possible to observe some BM material deposited beneath migrating hemocytes by TEM (Figure 2E), it was difficult to examine the dynamics of Col IV deposition beneath hemocytes by standard confocal microscopy due to the low level of fluorescence and small size of the deposits. We therefore utilized lattice light-sheet microscopy, which allows for enhanced spatiotemporal resolution with reduced phototoxicity [26]. Indeed, hemocyte motility within the ventral hemocoel was highly amenable to lattice light-sheet imaging at early stages of hemocyte dispersal with minimal photobleaching (Movie S3, part 1).

Imaging by lattice light-sheet microscopy revealed that, at the stage when hemocytes are aligned on the ventral midline, Col IV is primarily localized beneath hemocytes on the surface of the nerve cord and in the segmentally spaced dorsoventral channels of the VNC (Figures 2F and 2G; Movie S3, part 2). Subsequently, when hemocytes left the midline and migrated laterally, they appeared to deposit Col IV in a local fashion leaving puncta of matrix that eventually developed into longer fibrils (Figures 2F and 2G; Movie S3, part 3). Additionally, simultaneous imaging of Col IV and the hemocyte actin cytoskeleton showed that Col IV colocalized with actin fibers within lamellae (Figure S2A), suggesting hemocyte secretion of Col IV may require release along actin fibers or that recently released Col IV is rapidly remodelled by hemocytes using their actin network. Indeed, tracking movements in the Col IV matrix at high temporal resolution by particle image velocimetry revealed strong regions of ECM deformation beneath hemocyte lamellae, suggesting hemocyte traction forces are being exerted on the developing BM (Figure S2B; Movie S3, part 4).

As time-lapse imaging suggested that hemocytes are “plastering” embryonic surfaces with Col IV, we hypothesized that hemocyte developmental dispersal may be a critical part of the BM deposition process. Hemocytes develop in the anterior of the embryo, and after stage 10 of embryogenesis they disperse within the hemocoel using a combination of external guidance cues [23] and contact inhibition of locomotion, resulting in an evenly tiled cellular distribution [24, 25]. We therefore examined how the timing of BM component production correlated with the dispersal of hemocytes. While LanA was expressed during initial stages as hemocytes migrated from their source in the head of the embryo, Col IV production lagged behind by approximately 5 hr (Movie S4, part 1). As the induction of Col IV expression occurred largely after hemocyte dispersal, this suggested that hemocyte spreading within the embryo might be a prerequisite for Col IV delivery.

**Figure 3:**
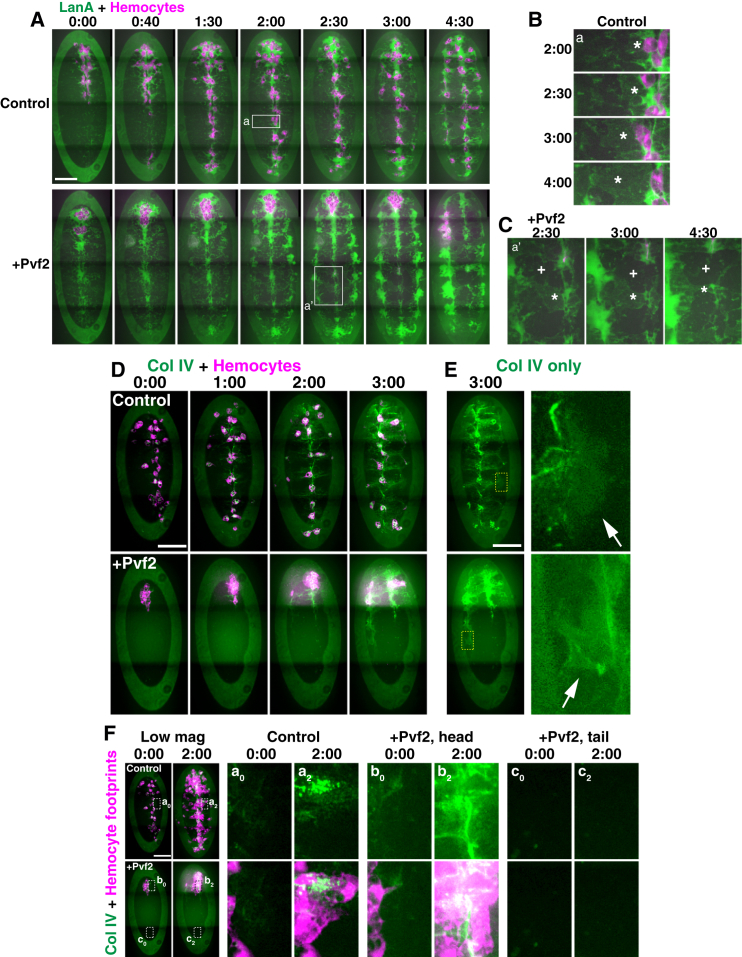
Hemocyte Dispersal Is Required for Even Delivery of Col IV throughout the Embryo (A) Time-lapse imaging of LanA distribution in control and Pvf2-overexpressing embryos, which leads to hemocyte clumping in the head. (B) High-magnification images from the regions highlighted by the rectangle in the control embryo in (A). A sheet-like structure containing Laminin (asterisk) extends laterally from the midline over time. (C) High-magnification images from the regions highlighted by the rectangle in the Pvf2-overexpressing embryo in (A). The Laminin sheet (asterisk) fails to extend, and the spaces not covered by Laminin (cross) enlarge over time. (D) Time-lapse imaging of Col IV distribution in control and Pvf2-overexpressing embryos. (E) Imaging of Col IV distribution at late stages revealing a diffuse distribution in the lateral hemocoel (arrows). Right panels are high-magnification images of the highlighted regions. (F) Hemocyte tracks or “footprints” were revealed by maximum-intensity projection of time-lapse images and correlated with Col IV localization. Right panels are high-magnification images of the highlighted regions showing Col IV localization (top panels) and Col IV localization with hemocyte footprints (bottom panels). Scale bars, 50 μm. Time points indicate time after the start of imaging (hr:min). See also Figure S3, Table S1, and Movie S4.

It was previously proposed that hemocytes were required for BM deposition specifically around the renal tubules during embryogenesis [16]; however, this was only interrogated in mutant embryos that were defective in both hemocyte migration and their survival [27]. To directly examine the role of hemocyte migration in BM component deposition, we caused aberrant hemocyte dispersal by misexpression of Pvf2, a platelet-derived growth factor (PDGF)-like chemotactic cue for hemocytes [23]. Overexpressing Pvf2 during hemocyte dispersal caused hemocytes to aggregate in the embryonic head (Figure 3A), which was likely due to a distraction of hemocytes from their normal Pvf source. LanA in wild-type embryos initially spread down the midline of the VNC, and this was unaffected by the inhibition of hemocyte migration (Figure 3A). Subsequently, in control embryos, a sheet-like structure containing Laminin extended from the middle of the VNC to lateral positions (Figures 3A and 3B; Movie S4, part 2). These nascent Laminin sheets were stable in time compared to the halos/trails of Laminin following migrating hemocytes, which fluctuated on the order of seconds (Movie S2, part 1). Therefore, we hypothesize that the extension of the Laminin sheets reflects the incorporation and growth of the polymerized matrix from a soluble source of Laminin residing predominantly on the midline. The initiation of Laminin incorporation was unaffected by Pvf2 overexpression (Figures 3A and 3C; Movie S4, part 2). However, in Pvf2-expressing embryos, the Laminin sheets failed to continue extending, leaving large gaps that increased in size by later stages of development (Figures 3A and 3C; Movie S4, part 2). This apparent breakdown of the Laminin matrix was similar to embryos lacking hemocytes (Figure S3A). Therefore, we speculated that Laminin produced by hemocytes may be critical for proper Laminin incorporation or that hemocyte movement, which opens up spaces between tissues (Figure 2D; Movie S2, part 1), could be aiding the growth of the Laminin matrix by enhancing its diffusion in the hemocoel. In contrast, despite an increase in Col IV upon Pvf2 overexpression (Figure S3B), confocal microscopy and lattice light-sheet imaging revealed that there was an uneven coverage of Col IV within the embryo, with most Col IV surrounding hemocytes in the head (Figures 3D–3F; Movie S4, parts 3 and 4). A similar local deposition of Col IV around hemocytes was also observed when hemocyte migration was disturbed by the expression of dominant-negative Rac (RacN17) or constitutively active Rac (RacV12) (Figures S3C and S3D). These results further suggest that Laminin deposition requires its diffusion within the embryonic hemocoel while Col IV is locally deposited by hemocytes.

While these data suggested a highly local mechanism of Col IV delivery by migrating hemocytes, a more complex picture emerged over longer time periods of imaging. At later stages of development, Col IV appeared to spread at a distance from hemocytes and fill the hemocoel (Figures 3D and 3E; Movie S4, part 3). We therefore imaged Col IV within embryos over a longer period of approximately 12 hr, which represents the time frame just prior to embryonic hatching. Inducing hemocyte aggregation in the anterior of the embryo through overexpression of Pvf2 or RacN17/RacV12 (Figures S3D and S3E) revealed an accumulation of Col IV around hemocytes approximately 6 hr after Col IV induction. However, by 12 hr the fluorescence of Col IV was distributed throughout the embryo despite a continued aggregation of hemocytes (Figures S3D and S3E; Movie S4, part 5). These data suggest that Col IV is eventually capable of spreading within the hemocoel but suffers from very slow effective diffusion.

**Figure 4:**
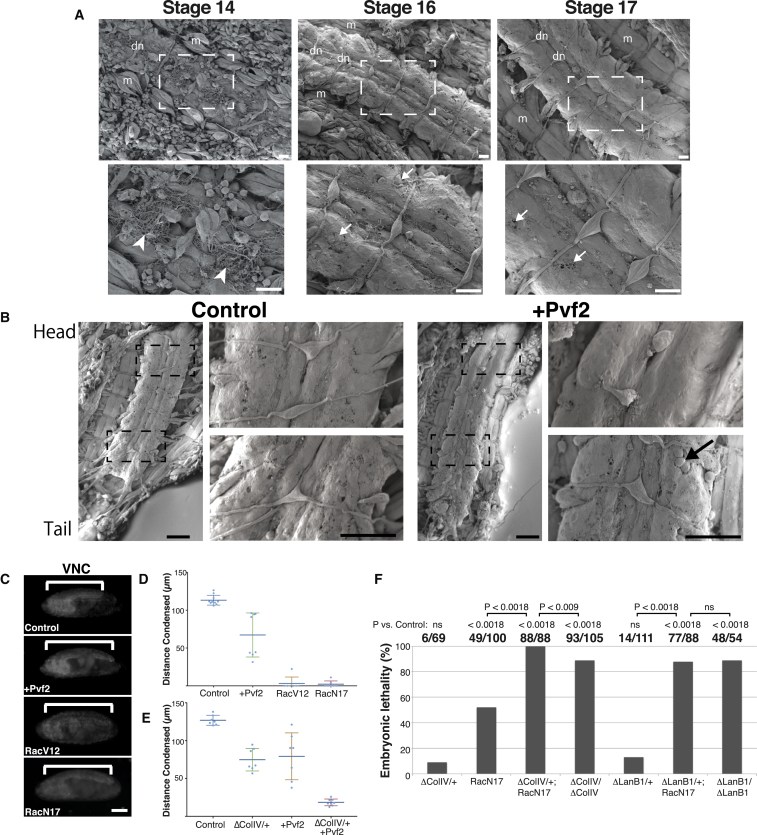
A Failure in Hemocyte Delivery of BM Leads to Morphogenetic Defects and Embryonic Lethality (A) Scanning electron microscopy of filleted embryonic VNC was performed to reveal the developing BM ensheathing the nerve cord. Lower panels show enlarged regions of the VNC highlighted in upper panels. Arrowheads, fibrils; arrows, BM holes; m, muscles; dn, dorsal nerves. Scale bars, 10 μm. (B) Scanning electron microscopy of the VNC from control and a Pvf2-overexpressing embryo. Enlarged images represent highlighted regions of the VNC from the head and tail regions, respectively. Arrow, BM hole. Scale bars, 20 μm. (C) Hemocyte migration was inhibited by overexpression of Pvf2, or hemocyte-specific expression of RacV12 or N17, and the VNC (brackets) was subsequently imaged using a glial-specific marker (RepoCherry). Scale bar, 100 μm. (D) Quantification of the distance the VNC condensed 8 hr after the start of the condensation process when hemocyte migration was inhibited by overexpression of Pvf2, or hemocyte-specific expression of RacV12 or N17. Bars indicate mean ± SD. (E) Quantification as in (D) in Col IV heterozygous mutants, Pvf2 overexpression, and embryos heterozygous for Col IV while simultaneously overexpressing Pvf2. Bars indicate mean ± SD. (F) The percentage of embryos that failed to hatch was quantified when causing aberrant hemocyte migration (RacN17), when hemocyte migration was combined with heterozygous BM mutants (ΔColIV and ΔLanB1), or in homozygous BM mutants. See also Figure S4, Table S1, and Movie S4.

We subsequently tested whether hemocyte migration and even BM deposition are functionally important for embryogenesis. We therefore examined VNC condensation, a known morphogenetic event that requires hemocytes and BM [28, 29]. As the BM surrounds the outer surface of the VNC, it is readily accessible to ultrastructural analysis. We therefore generated fillet preparations of the embryonic VNC, and we examined the developing BM by scanning electron microscopy (SEM). At stage 14 of development, the matrix surrounding the VNC was surprisingly fibrillar in appearance. However, by stage 15 these matrix fibrils were rapidly remodelled into a contiguous sheet containing holes that progressively closed during VNC condensation (Figure 4A). We next examined the distribution of the BM surrounding the VNC after inhibition of hemocyte migration, which severely affected the condensation process and led to a reduced embryonic viability (Figures 4B–4D and S4A). This revealed that, while the wild-type VNC showed a relatively even distribution of BM, Pvf2 overexpression led to a dense matrix in the head region with a sparse matrix surrounding the VNC in the tail (Figure 4B). This highlights that uniform hemocyte dispersal is indeed essential for even incorporation of BM and that the catching up in fluorescence levels upon the inhibition of hemocyte migration is likely the result of diffusing Col IV within the hemocoel rather than proper incorporation.

We also examined whether the severity of hemocyte migration defects correlated with embryonic lethality. As previously reported [30], hemocytes are completely essential for embryogenesis, as killing off hemocytes led to 100% lethality as measured by the frequency of embryonic hatching (Figure S4A). We next tested varying degrees of hemocyte migration defects. Expression of a dominant-negative Myosin II specifically in hemocytes led to minor clumping defects but no obvious effects on embryonic lethality (Figure S4A). In contrast, Pvf2 overexpression or hemocyte-specific expression of RacN17 led to intermediate migration defects and resulted in approximately 50% embryonic lethality (Figure S4A). Finally, hemocyte-specific expression of RacV12, which induced severe migration defects with hemocytes failing to disperse from their origin in the head, led to the most severe embryonic phenotype with 96% lethality (Figure S4A). Importantly, these differences in lethality were not correlated with levels of Col IV expression (Figures S3B and S4B), indicating that the lethality was not related to a change in Col IV levels. These data show that hemocyte migration is indeed essential for embryonic viability.

Finally, we examined whether a genetic interaction could be observed between hemocyte migration defects and BM mutant alleles. Causing aberrant hemocyte migration in the presence of a heterozygous *colIV* mutant allele, which led to a 50% reduction in Col IV expression (Figure S4C), abolished VNC condensation (Figure 4E) and induced a synergistic effect on embryonic lethality with 100% of embryos failing to hatch (Figure 4F). This lethality was higher than homozygous *colIV* mutants, showing that the synergy between hemocyte migration and Col IV reduction is not simply the result of a loss of Col IV expression; it also suggests that uneven Col IV deposition may be worse for the embryo than a complete loss of Col IV. In contrast, combining hemocyte migration defects with heterozygous *laminin* mutants led to a slight increase in lethality, which was similar to homozygous *laminin* mutant embryos (Figure 4F). These data further show that Col IV deposition is more dependent on hemocyte migration than other BM components, such as Laminin.

Here we show that during *Drosophila* embryogenesis, a subset of BM components requires local deposition by migrating hemocytes. This highlights that the ability of hemocytes to evenly spread throughout the embryo [24, 25] is part of a wider mechanism to uniformly deliver ECM. Therefore, as is increasingly realized for vertebrate macrophages, which are also involved in morphogenetic processes that involve matrix remodelling [31], hemocytes have important non-immune roles critical for development. Interestingly, mammalian macrophages have recently been revealed to produce various ECM components [32, 33, 34, 35, 36]; along with our data, this suggests that a critical role for macrophage-derived ECM may be more ubiquitous than previously recognized.

It is unclear why embryonic BM components like Col IV require local delivery by hemocytes, while in larvae they are thought to diffuse from the fat body [9]. This may be related to physiological differences between embryo and larva. In larvae, the heart pumps hemolymph around the animal, which may aid in the spreading of BM proteins. In contrast, the embryonic heart does not begin beating until stage 17 [37], which is after the start of Col IV deposition; in lieu of flowing hemolymph, BM factors with low effective diffusion may therefore require a moving source. Interestingly, recent work has revealed that at least one larval tissue, the developing ovary, requires hemocyte-specific production of Col IV [38], and it is possible that tissues not in direct contact with hemolymph require other mechanisms of BM deposition. However, it is unclear whether hemocytes associated with the ovary plaster Col IV in a manner similar to embryonic hemocytes or shed soluble Col IV similarly to the larval fat body [9].

It is also likely that there are differences between the mechanisms of *de novo* BM formation in the embryo versus homeostatic mechanisms involved in BM growth in the larva; when Col IV is first deposited in the embryo, its binding sites in the nascent Laminin matrix will be completely unsaturated leading to its rapid capture, thus preventing it from spreading far from its source. As Col IV saturates the BM at later stages of development, this would allow for its subsequent long-distance diffusion in older embryos and larvae. The larva may also have specific mechanisms that aid in Col IV solubility. Indeed, Sparc mutant larvae have abnormal extracellular BM deposits, and recent data from both *Drosophila* and *C. elegans* suggest that Sparc is a carrier for components like Col IV [9, 39, 40]. It is interesting to note that there is no embryonic phenotype in *Drosophila* in the absence of Sparc [39], suggesting that embryonic Col IV does not need to be solubilized, which we hypothesize is due to its specific hemocyte-dependent mechanism of delivery during *de novo* BM formation.

## STAR★Methods

### Key Resources Table

**Table:** 

REAGENT or RESOURCE	SOURCE	IDENTIFIER
**Experimental Models: Organisms/Strains**		

*D. melanogaster: w*^*1118*^	Bloomington Drosophila Stock Center	3605
*D. melanogaster:* Vkg (Col IV)-GFP	[12]	N/A
*D. melanogaster:* Trol (Perl)-GFP	Kyoto Stock Center	110836
*D. melanogaster:* LanA-GFP	[14]	N/A
*D. melanogaster:* LanB1-GFP	[14]	N/A
*D. melanogaster: srp*^*AS*^	[19]	N/A
*D. melanogaster: lanA*^*9-32*^	[41]	N/A
*D. melanogaster: lanA*^*MB01129*^	Bloomington Drosophila Stock Center	23555
*D. melanogaster:* Df(2L)LanB1 (ΔLanB1)	[42]	N/A
*D. melanogaster:* Df(2L)BSC172 (ΔCol IV)	Bloomington Drosophila Stock Center	9605
*D. melanogaster: trol*^*null*^	[43]	N/A
*D. melanogaster: srp*HemoGAL4	[27]	N/A
*D. melanogaster:* sn-Gal4	[44]	N/A
*D. melanogaster:* e22c-Gal4	[45]	N/A
*D. melanogaster: srp*-3xmCherry	This paper	N/A
*D. melanogaster:* UAS-Rpr	Gift from M. Dionne	N/A
*D. melanogaster:* UAS-RacN17	Bloomington Drosophila Stock Center	6292
*D. melanogaster:* UAS-RacV12	Bloomington Drosophila Stock Center	6291
*D. melanogaster:* UAS-RedStinger	Bloomington Drosophila Stock Center	8546 and 8547
*D. melanogaster:* UAS-LifeAct	[44]	N/A
*D. melanogaster:* UAS-mCherry-Moesin	[46]	N/A
*D. melanogaster:* UAS-Pvf2	[47]	N/A
*D. melanogaster:* UAS-secrGFP	[48]	N/A
*D. melanogaster:* UAS-ZipDN-GFP	[49]	N/A

**Oligonucleotides**		

Primer: 5′-CGAGGTCGACTCTAGAAAAT TTTGATGTTTTTAAATAGTCTTATCAGCA ATGGCAA-3′	This paper	N/A
Primer: 5′-ACGAAGCTTCTCTAGATAT GGGATCCGTGCTGGGGTAGTGC-3′	This paper	N/A

**Recombinant DNA**		

Plasmid: pJJH1295	Addgene	36914
Plasmid: pCaSpeR4	Gift from L. Ringrose	N/A
Plasmid: DSPL172 (encoding srp-3xmCherry)	This paper	N/A

**Software and Algorithms**		

LAS AF	Leica	http://www.leica-microsystems.com/home/
Volocity	PerkinElmer	http://cellularimaging.perkinelmer.com/downloads/
Zen	Carl Zeiss	https://www.zeiss.com/microscopy/int/products/microscope-software/zen.html
ImageJ/Fiji	Fiji	http://fiji.sc/
MATLAB	MathWorks	https://www.mathworks.com/products/matlab.html
Photoshop	Adobe	http://www.adobe.com/uk/products/photoshop.html?sdid=KKQPG&mv=search&s_kwcid=AL!3085!3!188961025881!e!!!!adobe%20photoshop&ef_id=WVDrMwAAAWGYhRWk:20170928120944:s
Illustrator	Adobe	http://www.adobe.com/uk/products/illustrator.html?sdid=KKQPG&mv=search&s_kwcid=AL!3085!3!176353364879!e!!!!adobe%20illustrator&ef_id=WVDrMwAAAWGYhRWk:20170928121009:s
Prism	GraphPad	https://www.graphpad.com/scientific-software/prism/
Excel	Microsoft	https://www.microsoft.com/en-gb/
Iterative Richardson-Lucy algorithm	[26]	N/A

**Other**		

10S Voltalef oil	VWR	24627.188
Lumox culture dish	Sarstedt	94.6077.305
M205 fluorescent dissection microscope	Leica	http://www.leica-microsystems.com/home/
LSM 880 confocal microscope	Carl Zeiss	https://www.zeiss.com/corporate/int/home.html
63x NA 1.4 Plan-Apochromat oil objective	Carl Zeiss	https://www.zeiss.com/corporate/int/home.html
Ultraview spinning disk	PerkinElmer	https://science.nichd.nih.gov/confluence/display/mic/Perkin-Elmer+Ultraview+RS
Lattice light-sheet micrcoscope (LLSM) in the Advanced Imaging Center (AIC) at the Howard Hughes Medical Institute Janelia research campus	[26]	https://www.janelia.org/open-science/advanced-imaging-center-aic
5 mm round glass coverslips	Warner Instruments	CS-5R
Diode lasers for LLSM	MPB Communications	http://www.mpbcommunications.com/
LLSM excitation objective (0.65 NA, 3.74-mm WD)	Special Optics	http://specialoptics.com/
LLSM detection objective (CFI Apo LWD 25XW, 1.1 NA)	Nikon	https://www.nikoninstruments.com/en_GB/Product-Selectors/Objective-Selector
sCMOS cameras for LLSM	Hamamatsu	Orca Flash 4.0 v2
200nm tetraspeck beads	Invitrogen	T7280
EM PACT2 high-pressure freezer	Leica	N/A
Leica AFS (automatic freeze substitution system)	Leica	N/A
SPURR resin	TAAB	ERL 4221D
FEI Tecnai 12 transmission electron microscope	EI Tecnai Family	N/A
16000M camera	AMT	N/A
JSM 7800prime scanning electron microscope	JEOL	N/A

### Contact for Reagent and Resource Sharing

Further information and requests for resources and reagents should be directed to and will be fulfilled by the Lead Contact, Brian Stramer (brian.m.stramer@kcl.ac.uk).

### Experimental Model and Subject Details

#### Fly stocks and preparation

*w*^*1118*^ were used as wild-type controls. Vkg/Col IV [12] and Trol/Perl [13] GFP-protein trap strains as well as LanA-GFP and LanB1-GFP fosmid transgenic lines [14] were used to visualize BM components. The following mutant alleles and deficiencies were used: *srp*^*AS*^ (lacking hemocytes), *lanA*^*9-32*^ [41], *lanA*^*MB01129*^ (Bloomignton *Drosophila* Stock Center/BDSC), Df(2L)LanB1 (removing *lanB1* [42], referred to as ΔLanB1), Df(2L)BSC172 (referred to as ΔCol IV, removing a chromosomal region including the both of the two *Drosophila* Col IV genes *vkg* and *Cg25C,* obtained from BDSC), and *trol*^*null*^ [43] (referred to as ΔPerl). The ‘ΔLanB1, ΔCol IV’ recombinant flies were maintained as a stock whose second chromosome carries the both deficiencies and the third chromosome harbors ectopic LanB1-GFP, which rescues the lethality caused by a genetic interaction between the LanB1 and Col IV mutations. The ‘Perl-GFP; ΔLanB1, ΔCol IV’ (Figure 1D) embryos were selected by the absence of the fluorescence from both the marker on second chromosome balancer and LanB1-GFP, which was distinguished by its earlier expression from Perl-GFP. The *srp*HemoGAL4 [27] (referred to as srp-Gal4) and sn-Gal4 [44] were used to express transgenes specifically in hemocytes. e22c-Gal4 [45] was used to widely express transgenes throughout various tissues including the epithelium surrounding embryo. *srp*-3xmCherry, whose construction is described in the following section, expresses a tandem trimer of mCherry under the direct control of the *srp*Hemo promoter sequence [27], thus labeling hemocytes in a Gal4-independent manner. RepoCherry (gift from Clemens Cabernard, University of Basel) expresses mCherry under the direct control of the repo promoter sequence, thus labeling glia in a fashion independent of Gal4. The following UAS lines were used: UAS-Rpr (a gift from Marc Dionne), UAS-RacN17, UAS-RacV12, UAS-RedStinger (BDSC), UAS-LifeAct [44], UAS-mCherry-Moesin [46], UAS-Pvf2 [47], UAS-secrGFP [48], UAS-ZipDN-GFP [49]. Flies were left to lay eggs on grape juice/agar plates overnight at 25°C. Embryos were dechorionated in bleach. Embryos of appropriate genotype were identified based on the presence of fluorescent probes and/or the absence of balancer chromosomes expressing fluorescent markers. The genotypes of the embryos used in each experiment are summarized in the Table S1.

### Method Details

#### Construction of srp-3XmCherry

A 2.5 kb XbaI-EcoRI fragment which contains 3 repeats of mCherry was cloned from pJJH1295 [50] (a gift from Jürgen Heinisch, Addgene plasmid #36914), into the multiple cloning site of pCaSpeR4 (a gift from Leonie Ringrose, IMBA, Vienna). Subsequently, a 4.3 kb fragment of the *srp* promoter was amplified from plasmid *srp*HemoA [27] (a gift from Katja Brückner) by PCR with the following primers: 5′-CGAGGTCGACTCTAGAAAATTTTGATGTTTTTAAATAGTCTTATCAGCAATGGCAA-3′ and 5′-ACGAAGCTTCTCTAGATATGGGATCCGTGCTGGGGTAGTGC-3′.

This fragment was cloned upstream of the 3XmCherry fragment at the XbaI site, by Infusion cloning to create DSPL172. The plasmid was sequenced; we detected some minor errors in the *srp* promoter fragment which may account for the maintenance of the expression in larvae and adults.

#### Widefield and confocal microscopy

For analysis of GFP expression levels of BM proteins, dechorionated embryos were mounted in 10S Voltalef oil (VWR) between a glass coverslip covered with heptane glue and a gas-permeable Lumox culture dish (Sarstedt) as described previously [51, 52]. For any other widefield and confocal analyses single embryos were mounted without heptane glue. Widefield images were acquired with an M205 fluorescent dissection microscope (Leica). The heatmap of GFP fluorescence was made by setting the lookup table to ‘Fire’ with ImageJ (http://imagej.nih.gov/ij/). For FRAP analyses, an LSM 880 confocal microscope (Carl Zeiss) equipped with a 63x NA 1.4 Plan-Apochromat oil objective was used. After taking control images, the regions of interest (N ≥ 3) were bleached 15 times with a 488 nm laser at 100% laser transmission with 16.38 μsec/pixel dwell time, immediately followed by acquisition of 95 series of images every 1.5 s. All other confocal images were taken on an Ultraview spinning disk (PerkinElmer) equipped with 63 × NA 1.4 Plan-Apochromat oil objective. Maximum projection images were made from approximately 10 μm Z stacks. If autofluorescence from the overlying vitelline membrane perturbed observation, the autofluorescence was manually erased before making maximum projection images. To image wider regions of the VNC of embryos by confocal microscopy, multiple neighboring spinning disk images were stitched using Volocity software (PerkinElmer). This caused artificial appearance of dark lines at the borders between the connected images due to uneven illumination by spinning disk microscopy. Image processing was done by using ImageJ and Photoshop (Adobe) in addition to Volocity.

#### Lattice light-sheet microscopy

The lattice light sheet microscope (LLSM) used in these experiments is housed in the Advanced Imaged Center (AIC) at the Howard Hughes Medical Institute Janelia research campus. The system is configured and operated as previously described [26]. Briefly, dechorionated embryos were attached to 5 mm round glass coverslips (Warner Instruments, Catalogue # CS-5R) with heptane glue and imaged in phosphate-buffered saline at room temperature. Samples were illuminated by lattice light-sheet using 488 nm or 560 nm diode lasers (MPB Communications) through an excitation objective (Special Optics, 0.65 NA, 3.74-mm WD). Fluorescent emission was collected by detection objective (Nikon, CFI Apo LWD 25XW, 1.1 NA), separated to two light paths using a dichroic mirror, and detected by sCMOS cameras (Hamamatsu Orca Flash 4.0 v2), respectively. Acquired data were deskewed as previously described [26], and deconvolved using an iterative Richardson-Lucy algorithm. Point-spread functions for deconvolution were experimentally measured using 200nm tetraspeck beads (Invitrogen, Catalogue # T7280) adhered to 5 mm glass coverslips for each excitation wavelength.

#### Particle image velocimetry

A 2D cross-correlation algorithm adapted from classical particle image velocimetry was implemented [25]. In brief, this method compares a region of interest in an image (source image) with a subframe of a subsequent image (search image). The sizes of the source and search regions are determined on the basis of the feature size to be tracked and the area of their expected displacement (i.e., Col IV-GFP puncta). For this analysis, source and search images encompassing areas of 1.5 μm^2^ and 2.5 μm^2^, respectively, were used. The whole search image was analyzed by computing a cross-correlation coefficient between the source image, and a sub-image of the search image shifted by one pixel. The displacement of the basement membrane was measured by finding the maximum coefficient within the resulting cross-correlation map. The analysis was performed using a temporal resolution of 6 s To filter anomalous tracking data, only displacements that had a cross-correlation coefficient above a certain threshold, c_0_, were kept. For the present work, the threshold was set at c_0_ = 0.3. Finally, a spatial convolution with a Gaussian kernel (variance of 1μm), and temporal convolution with temporal kernel of 25 s were used to interpolate the measured displacements to cover all the pixels within the cell outline. The complete algorithm for this analysis was implemented in MatLAB (MathWorks®).

#### Transmission electron microscopy

Dechorionated embryos at stage 15 were placed in specimen planchets containing a cryoprotectant mix of 30% Dextran and 0.1% Triton X-100. Planchets were then high-pressure frozen using the Leica EM PACT2 high-pressure freezer (Leica Microsystems) and stored in liquid nitrogen until freeze-substitution (FS). The FS protocol followed in this paper is based on the previously reported quick method [53] with minor modifications. In summary, frozen samples were transferred from liquid nitrogen to pre-cooled (−130°C) FS medium (1% Osmium tetroxide, 0.1% uranyl acetate in acetone) and freeze-substituted using the Leica AFS, automatic freeze substitution system (Leica Microsystems). Initial FS was carried out over a period of 5 hr from −130°C to −15°C. The samples were then removed from the AFS in metal canisters and allowed to reach room temperature on their own. Once at room temperature, samples were thoroughly washed in acetone for 40 min before infiltrated and embedded in SPURR resin (TAAB). Ultrathin sections (50-70 nm) were prepared using a Reichert-Jung Ultracut E ultramicrotome, mounted on 150 mesh copper grids and contrasted using uranyl acetate and lead citrate. Samples were examined on a FEI Tecnai 12 transmission microscope operated at 120 kV. Images were acquired with an AMT 16000M camera.

#### Scanning electron microscopy

Embryos were prepared as previously described [54, 55] with some modification. Briefly, after dechorionation with bleach, live embryos at stage 14-17 were manually taken out of the vitelline membrane using an insect pin and attached to a glass coverslip covered with heptane glue with the dorsal side up. The embryos were then filleted in phosphate-buffered saline to expose the dorsal surface of the VNC. Subsequently the embryos were fixed for 45 min at room temperature with 4% (w/v) formaldehyde, and further fixed with 2.5% (v/v) glutaraldehyde in 0.1M cacodylate buffer (pH 7.2) overnight at 4°C. In order to minimize shrinking/cracking artifacts during processing, osmium tetroxide was omitted from the protocol. Instead, samples were stained for 1 hr with 0.1% (w/v) aqueous tannic acid, and 20 min with 0.2% (w/v) aqueous uranyl acetate. Samples were thoroughly washed between treatments. Finally, embryos were dehydrated, critically point dried and sputter coated with 4nm gold for SEM. Images were acquired on a JEOL JSM 7800prime scanning electron microscope operated in Gentle Beam mode to reduce radiation damage and surface charging. Samples were imaged using a gun voltage of 2.6-3 kV. A negative bias of 2kV was applied to the sample stage to decelerate incident electrons, which resulted on a landing voltage on the sample of 0.6–1 kV.

#### Quantification of GFP fluorescence

Using ImageJ, the average fluorescence intensity in each individual GFP-expressing embryo at each single time point until hatching was measured. These values were sums of GFP fluorescence and autofluorescence of the embryo. From each value, the mean intensity of embryos not expressing GFP (N ≥ 5 at time zero) at the same time point of development was subtracted to obtain the intensity of GFP fluorescence. The resultant mean ± SEM of values was plotted against time. To determine the maximum expression level of each individual embryo, the highest GFP intensity for each individual embryo was determined.

#### Hemocyte footprints

For each time point t, the temporal maximum intensity projection of all the hemocyte images through time zero to t, taken every 15 min, was created with ImageJ.

#### Lethality assay

Embryos of appropriate genotypes older than stage 15 were selected and incubated on grape juice agar overnight at room temperature. Subsequently, the number of embryos that failed to hatch were quantified.

### Quantification and Statistical Analysis

Excel (Microsoft) and Prism (Graphpad) were used for drawing graphs and statistical analyses. In column scatterplots, bars indicate median and interquartile range. Line plots show the mean ± SEM of all the individual data from repeated experiments unless otherwise stated. For statistical analyses, the data shown in column scatterplots were examined by the Mann-Whitney test. Contingency tables of embryonic lethality were analyzed by the Fisher’s exact test. If necessary the Bonferroni correction for multiple comparisons were carried out.

## Author Contributions

Y.M.D.E.S., and B.M.S. designed the experiments. Y.M., A.L., A.D., B.J.S.-S., E.S.-M., L.Y., A.G., G.V., R.A.F., J.M.H., T.-L.C., D.E.S., and B.M.S. generated reagents and performed experiments. Y.M., B.J.S.-S., L.Y., D.E.S., and B.M.S. wrote the manuscript.

## Supplemental Information

Document S1. Figures S1–S4 and Table S1

Movie S1. Live Imaging of BM Reporter Induction, Related to Figure 1 Time-lapse movie of fluorescently tagged basement membrane components during embryogenesis revealing the temporal hierarchy of component expression.

Movie S2. Confocal Imaging of Hemocyte Migration and Laminin/Col IV Expression, Related to Figure 2 Part 1 (00:00-00:26) with Laminin. Time-lapse movie of LanA-GFP (green) and hemocytes (magenta) overlying the ventral nerve cord using standard confocal microscopy. Note the halos of Laminin surrounding hemocytes and the trails of Laminin following hemocytes as they migrate laterally, which represents Laminin diffusing within the hemocoel. Timestamp: minutes:seconds. Part 2 (00:27-end) with Col IV. Time-lapse movie of Col IV-GFP (green) and hemocytes (magenta) overlying the ventral nerve cord using standard confocal microscopy. Note that Col IV fluorescence is mainly associated with the ventral nerve cord and does not fill the hemocoel. Timestamp: hours:minutes:seconds.

Movie S3. Lattice Light-Sheet Movies of Hemocyte Migration and Col IV Deposition, Related to Figures 2 and S2 Part 1 (00:00-01:40). Lattice light-sheet movie of hemocyte migration. Lattice light-sheet imaging of hemocytes containing labelled F-actin (green) and a cytoplasmic marker (magenta) during their initial migration from the ventral midline during Stage 14. The midline is aligned horizontally along the bottom of the image and anterior of the embryo is to the left. Time-lapse images were acquired every 2 seconds. Movie is played at the rate of 25 frames per second. Part 2 (01:40-02:00). 3D reconstruction of Hemocytes and Col IV distribution. 3D-reconctruction of Lattice light-sheet images of Col IV-GFP (green) and hemocytes (magenta) during their initial migration from the ventral midline during Stage 14. At this stage Col IV is localized along the midline (arrowhead) and in segmentally spaced channels that run through the nerve cord (arrows). Part 3 (02:00-02:38). Lattice light-sheet movie of hemocyte deposition of Col IV. Note that during hemocyte migration from the midline, Col IV is deposited in a local fashion beneath hemocyte lamellae. As hemocytes migrate laterally from the midline they deposit puncta of Col IV (arrows), which eventually develop into longer fibrils (arrowheads). Timestamp-minutes: seconds. Part 4 (02:38-end). Lattice light-sheet movie of hemocyte movement and Col IV matrix deformation. Lattice light-sheet imaging of Col IV-GFP (green) and hemocyte actin (magenta). Time-lapse images were acquired every 3 seconds. The right panel shows tracking of Col IV features by particle image velocimetry (PIV) over a temporal resolution of 6 seconds, which reveals rapid contractile movement in the developing basement membrane. The heatmap of the PIV (arbitrary units: red- high, blue-low) is overlaid onto the Col IV-GFP image. Timestamp: minutes:seconds.

Movie S4. Correlation of BM Protein Localization and Hemocyte Dispersal, Related to Figures 3, 4, and S3 Part 1 (00:00-00:26). Time-lapse movie of hemocyte dispersal versus Col IV and Laminin expression levels. Time-lapse movie of LanA-GFP and Col IV-GFP over time during development while also comparing with the developmental dispersal of hemocytes. The fluorescence of BM components is displayed both in grayscale and as a heatmap. The graph highlights the normalised fluorescence intensity of BM components throughout embryogenesis. Note that LanA is expressed early during the dispersal process. In contrast Col IV expression lags behind and is induced largely after hemocytes spreading throughout the embryo. Timestamp: hours:minutes. Part 2 (00:26-00:38). Time-lapse movie of Laminin spreading in control versus Pvf2 overexpressing embryos. Time-lapse movie of LanA-GFP (green) and hemocytes (magenta) overlying the ventral nerve cord using standard confocal microscopy in a control (left panel) and a Pvf2 overexpressing embryo (right panel). Timestamp: hours:minutes. Part 3 (00:38-00:55). Time-lapse movie of Col IV spreading in control versus Pvf2 overexpressing embryos. Time-lapse movie and 3D reconstruction of Col IV-GFP (green) and hemocytes (magenta) overlying the ventral nerve cord using standard confocal microscopy in a control (left panel) and a Pvf2 overexpressing embryo (right panel). Timestamp: hours:minutes. Part 4 (00:55-01:11). Lattice light-sheet movie of hemocyte hemocyte migration and Col IV deposition in a Pvf2. Lattice light-sheet imaging of Col IV-GFP (green) and hemocytes (magenta) upon overexpression of Pvf2 ubiquitously in the embryo. Note that most of the Col IV is concentrated in the anterior part of the embryo (anterior is up) beneath the hemocytes. Timestamp: minutes:seconds. Part 5 (01:11-end). Time-lapse movie of hemocyte dispersal and Col IV expression in a control versus a Pvf2 expressing embryo. Time-lapse movie of Col IV-GFP (green) and hemocytes (magenta) in a Control embryo versus an embryo overexpressing Pvf2, which concentrates hemocytes in the head. Note that in the Control, Col IV expression is induced relatively evenly throughout the embryo. In contrast, when hemocytes are concentrated in the head, Col IV is initially unevenly released and primarily concentrated around hemocytes. However, despite the continued aggregation of hemocytes, by the end of embryogenesis Col IV has spread throughout the embryo. Timestamp: hours:minutes.

Document S2. Article plus Supplemental Information
