# Supplementary material for: A Moving Source of Matrix Components Is Essential for De Novo Basement Membrane Formation
Source: Curr Biol. 2017 Nov 20;27(22):3526–3534.e4. doi: 10.1016/j.cub.2017.10.001 (PMC5714436; doi:10.1016/j.cub.2017.10.001)
Supplement: Document S1. Figures S1–S4 and Table S1 [file mmc1.pdf]

Current Biology, Volume *27*

## Supplemental Information

# A Moving Source of Matrix Components Is Essential for *De Novo* Basement Membrane Formation

**Yutaka Matsubayashi, Adam Louani, Anca Dragu, Besaiz J. Sánchez-Sánchez, Eduardo Serna-Morales, Lawrence Yolland, Attila Gyoergy, Gema Vizcay, Roland A. Fleck, John M. Heddleston, Teng-Leong Chew, Daria E. Siekhaus, and Brian M. Stramer**

# Figure S1

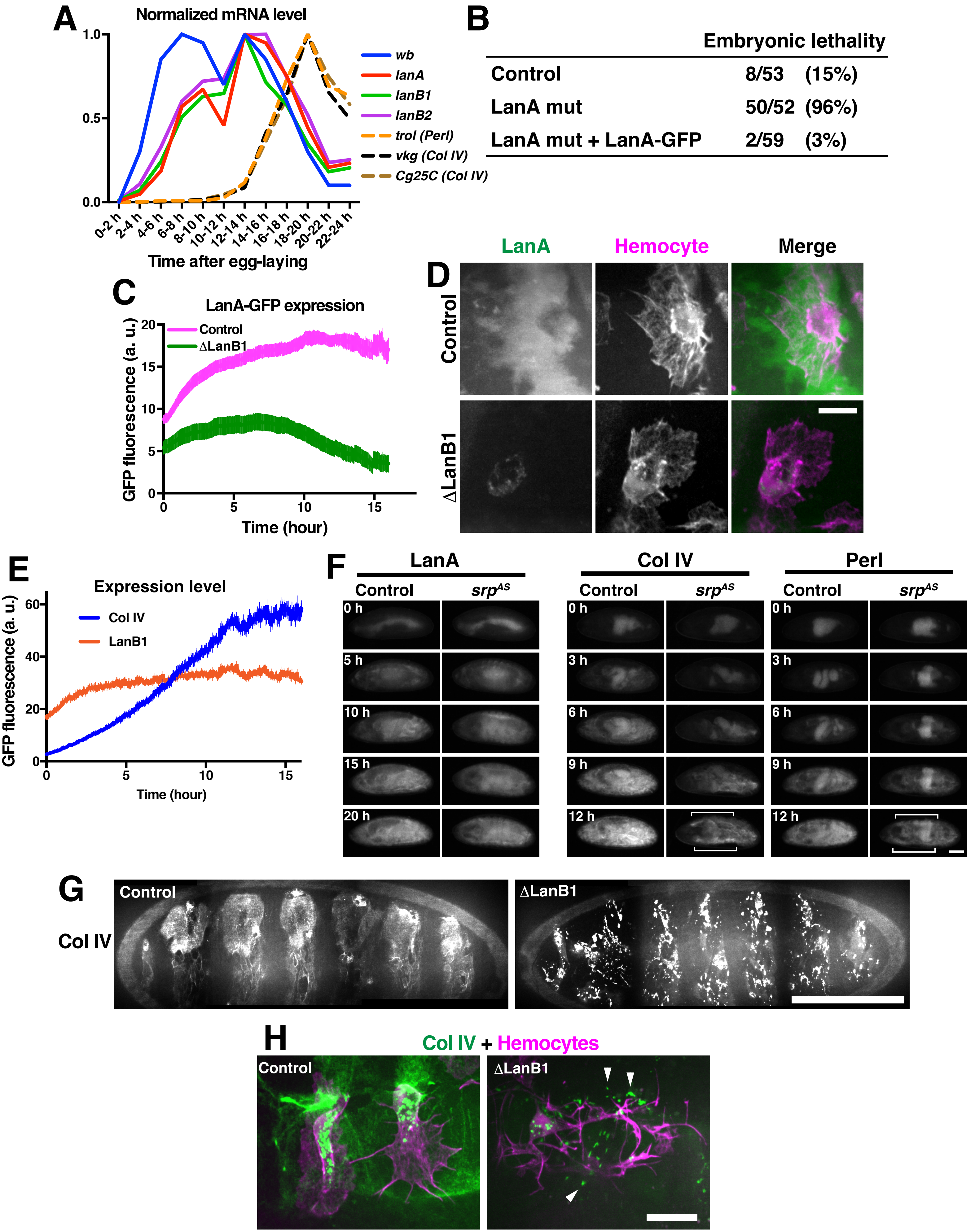
A
Normalized mRNA level
1.0
0.5
0.0
0-2 h
2-4 h
4-6 h
6-8 h
8-10 h
10-12 h
12-14 h
14-16 h
16-18 h
18-20 h
20-22 h
22-24 h
Time after egg-laying
wb
lanA
lanB1
lanB2
trol (Perl)
vkg (Col IV)
Cg25C (Col IV)
B
Embryonic lethality
Control 8/53 (15%)
LanA mut 50/52 (96%)
LanA mut + LanA-GFP 2/59 (3%)
C
LanA-GFP expression
Control
ΔLanB1
GFP fluorescence (a. u.)
Time (hour)
D
LanA
Hemocyte
Merge
Control
ΔLanB1
E
Expression level
Col IV
LanB1
GFP fluorescence (a. u.)
Time (hour)
F
LanA
Col IV
Perl
Control
srpAS
0 h
3 h
5 h
6 h
9 h
10 h
12 h
15 h
20 h
G
Control
ΔLanB1
Col IV
H
Col IV + Hemocytes
Control
ΔLanB1

# Figure S2

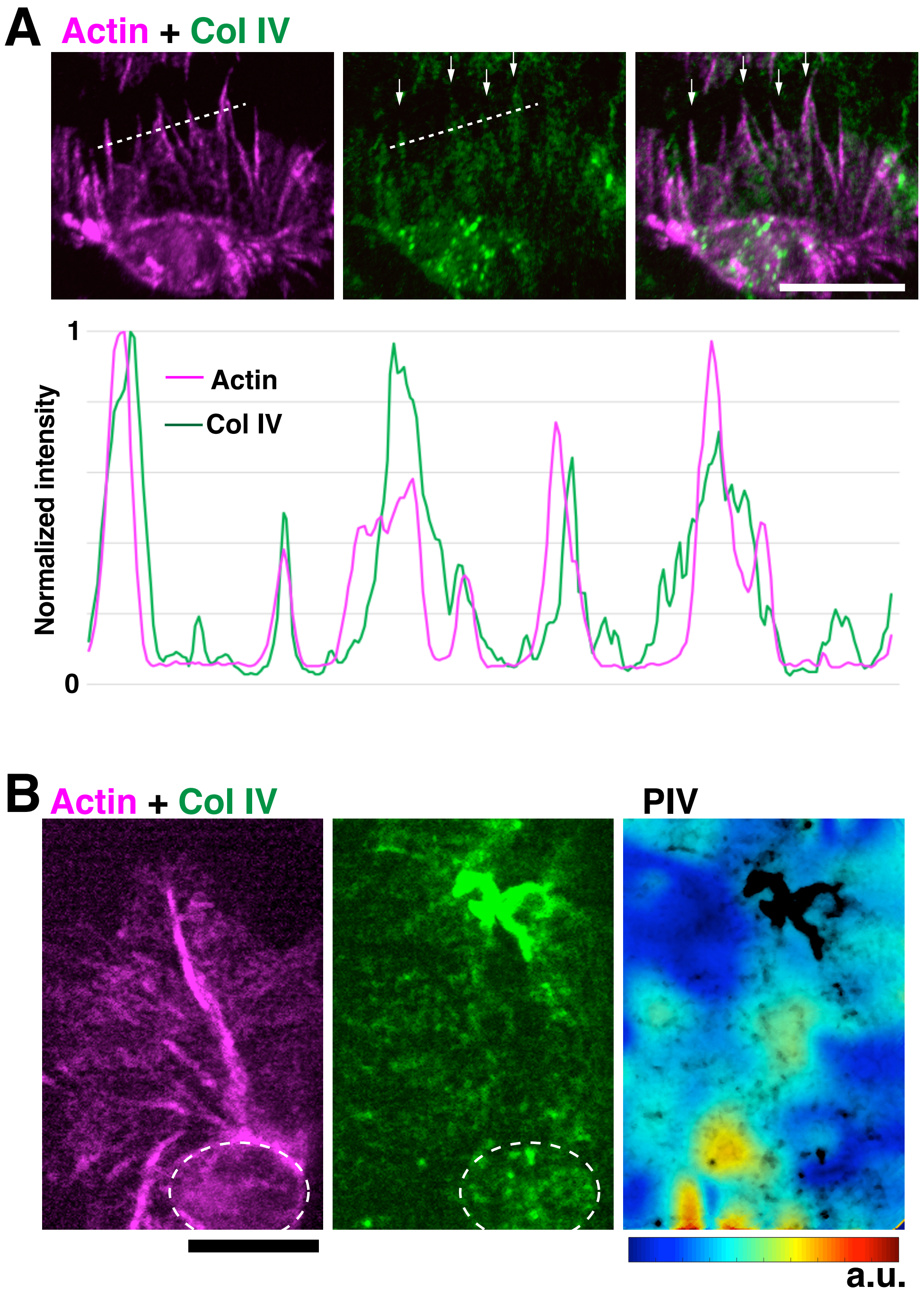
A
Actin + Col IV
1
0
Normalized intensity
Actin
Col IV
B
Actin + Col IV
PIV
a.u.

# Figure S3

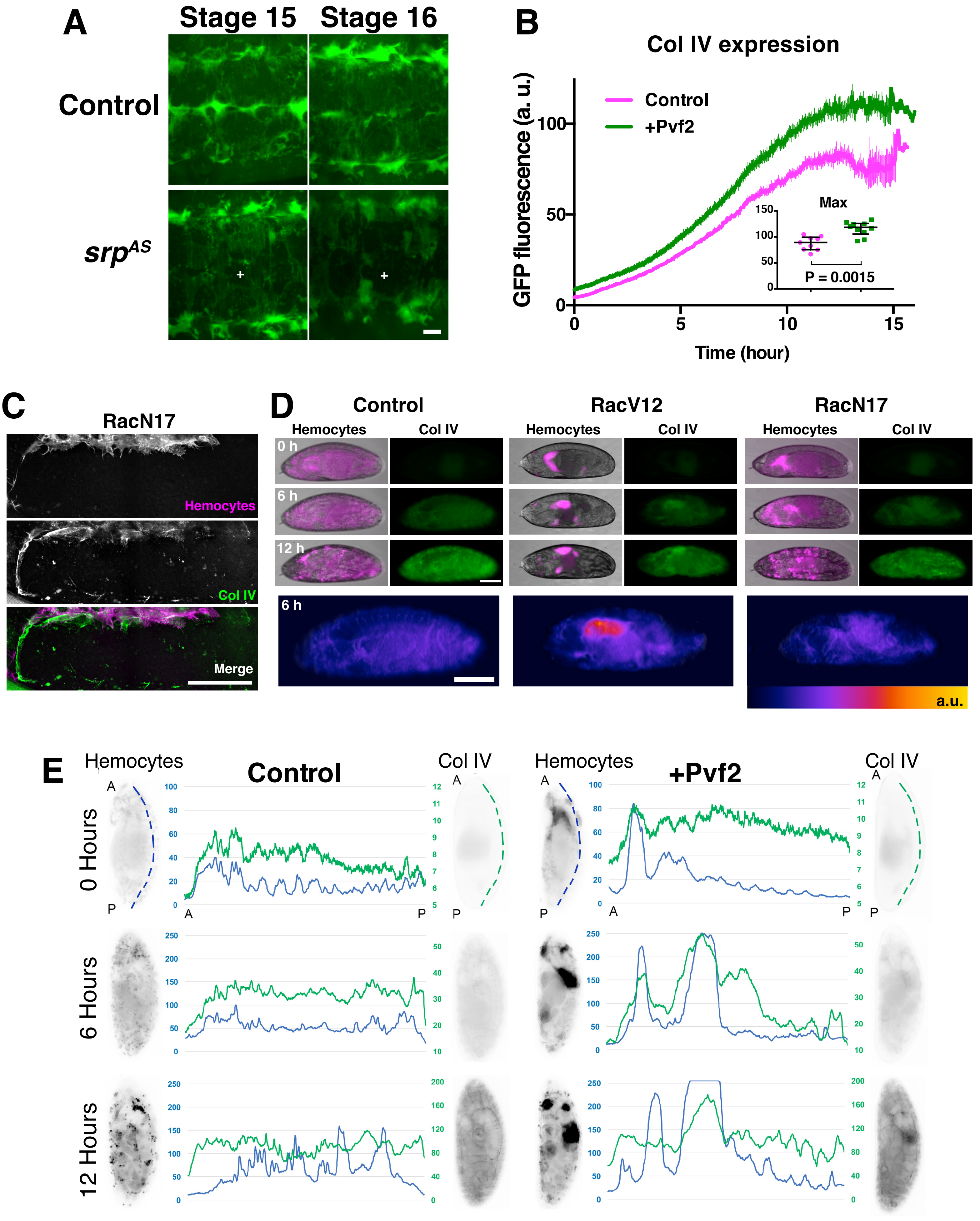

A
Stage 15
Stage 16
Control
srpAS
B
Col IV expression
Control
+Pvf2
GFP fluorescence (a. u.)
Time (hour)
Max
P = 0.0015
C
RacN17
Hemocytes
Col IV
Merge
D
Control
RacV12
RacN17
Hemocytes
Col IV
0 h
6 h
12 h
a.u.
E
Hemocytes
Control
Col IV
+Pvf2
0 Hours
6 Hours
12 Hours
A
P

# Figure S4

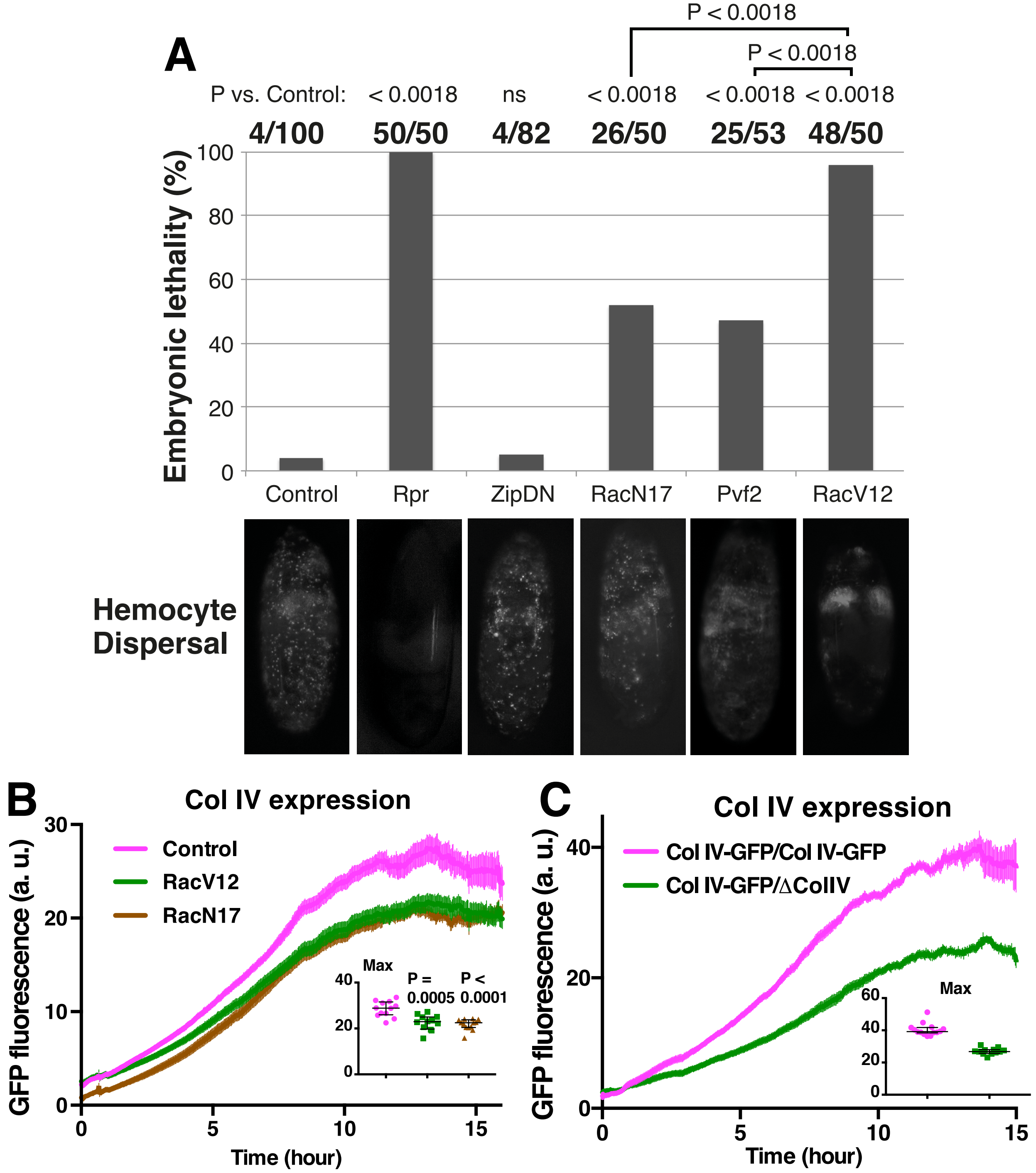

A
P < 0.0018
P < 0.0018
P vs. Control: < 0.0018 ns < 0.0018 < 0.0018 < 0.0018
4/100 50/50 4/82 26/50 25/53 48/50
Embryonic lethality (%)
100
80
60
40
20
0
Control
Rpr
ZipDN
RacN17
Pvf2
RacV12
Hemocyte Dispersal
B
Col IV expression
Control
RacV12
RacN17
GFP fluorescence (a. u.)
Time (hour)
Max
P = 0.0005
P < 0.0001
C
Col IV expression
Col IV-GFP/Col IV-GFP
Col IV-GFP/ΔColIV
GFP fluorescence (a. u.)
Time (hour)
Max

**Figure S1. Characterization of the levels and localization of BM components. Related to Figure 1.**

**(A)** Developmental time-course of mRNA expression levels encoding the BM proteins. The RNAseq data was downloaded from modENCODE. Note that the expression of Laminin subunits (*wb, lanA, lanB1 and lanB2*, solid lines) precedes that of Collagen IV (*vkg* and *Cg25c*) and Perlecan (*trol*) (dashed lines).

**(B)** LanA-GFP rescues LanA mutant. Lethality of the embryos was quantified in the indicated genotypes. For each genotype, the results are shown in the format of (number of dead embryos) / (total number of embryos examined).

**(C)** Expression of LanA-GFP depends on LanB1. The fluorescence levels of LanA-GFP in Control (magenta) and ΔLanB1 (green) embryos was measured and plotted against time as in Figure 1C. Mean ± SEM.

**(D)** Confocal images of LanA-GFP (green) and hemocytes (magenta) in Control and ΔLanB1 embryos. Note that LanA-GFP is reduced and remaining LanA is not found outside hemocytes in ΔLanB1 embryos. Scale bar, 10 μm.

**(E)** The fluorescence levels of Col IV-GFP (blue) and LanB1-GFP (orange) was measured and plotted against time as in Figure 1C. Note that expression of LanB1-GFP precedes that of Col IV-GFP. Mean ± SEM.

**(F)** Time-lapse imaging of *Drosophila* embryos expressing GFP-tagged BM components in Control and *srp*$^{AS}$ mutant embryos, in which hemocytes fail to develop. Note that due to its earlier expression, LanA imaging was started at embryonic stage 12, while Col IV and Perl imaging was started at stage 15. Scale bar, 100 μm. Brackets highlight fat body expression.

**(G)** Confocal images of Col IV in control and *lanB1* mutant (ΔLanB1) embryos. Note the large disorganised aggregations in the ΔLanB1 mutant. Scale bar, 100 μm.

**(H)** Confocal images of Col IV (green) and hemocytes (magenta) in Control and a *lanB1* mutant (ΔLanB1). Note that the Col IV aggregations (arrowheads) are outside hemocytes in the ΔLanB1 mutant. Scale bar, 10 μm.

**Figure S2. Colocalization of actin with newly deposited Col IV and tracking BM deformation.**
**Related to Figure 2.**

**(A)** Lattice light-sheet image of Col IV (green) and hemocyte actin (magenta). Graph highlights a linescan of Col IV and Actin revealing their colocalization (arrows).

**(B)** Time-lapse imaging by lattice light sheet microscopy of Col IV (green) and hemocyte actin (magenta). The right panel shows tracking of Col IV features by particle image velocimetry (PIV), which reveals rapid movements surrounding hemocyte lamellae in the developing basement membrane. Note the strong intracellular vesicles of Col IV within the hemocyte cell body (circle) with the remainder of the Col IV representing extracellular deposited protein. The heatmap is in arbitrary units and overlaid onto the Col IV image. Scale bars, 10 μm.

**Figure S3. Examination of the distribution of BM components upon hemocyte perturbation.**
**Related to Figure 3.**

**(A)** Localization of LanA in control and *Srp*$^{AS}$ mutant embryos, which lack hemocytes. In the *Srp*$^{AS}$ embryos, which lack hemocytes, gaps in the nascent Laminin sheet enlarge over time (cross). Scale bar, 10 μm.

**(B)** The fluorescence of Col IV-GFP in Control (magenta) and embryos overexpressing Pvf2 (green) was measured and plotted as in Figure 1C. Note that Pvf2 overexpression enhances the expression of Col IV-GFP. Mean ± SEM. Inset shows quantification of the maximum fluorescence in each single embryo. Bars in the inset indicate median ± interquartile range.

**(C)** Confocal images of hemocytes (magenta) and Col IV (green) in an embryo expressing RacN17 specifically in hemocytes. Col IV is mainly deposited around the aggregated hemocytes. Scale bar, 50 μm.

**(D)** Comparison of hemocyte developmental dispersal with expression levels of Col IV-GFP by widefield microscopy when hemocyte migration is affected by hemocyte-specific expression of RacV12 or RacN17. Bottom panels are heatmaps of Col IV expression. Scale bars, 100 μm.

**(E)** Comparison of hemocyte developmental dispersal with expression levels of Col IV-GFP by widefield microscopy in control and embryos overexpressing Pvf2. Graphs represent linescans of hemocytes (blue) and Col IV (green) within the highlighted regions.

Imaging was started at embryonic stage 12, which is prior to the induction of Col IV expression. Scale bar, 100 μm.

**Figure S4. Quantification of embryonic lethality and Col IV expression levels upon hemocyte perturbation**
**Related to Figure 4.**

**(A)** Hemocytes were killed off by hemocyte-specific expression of an apoptosis inducing protein, Reaper (Rpr). Alternatively, hemocyte migration was impaired by hemocyte-specific expression of dominant negative myosin II (ZipDN), dominant negative Rac (RacN17), constitutively active Rac (RacV12), or ubiquitous expression of Pvf2, and embryonic hemocyte distribution imaged by widefield microscopy. The percentage of embryos that failed to hatch in each genotype was quantified. Scale bar, 100μm.

**(B)** The fluorescence of Col IV-GFP in Control embryos (magenta) and those expressing RacV12 (green) or RacN17 (brown) in hemocytes was measured and plotted as in Figure 1C. Note that while RacN17 and RacV12 decrease Col IV levels to a similar extent, they are statistically different with regards to inducing embryonic lethality (see Figure S4B). Mean ± SEM. Inset shows quantification of the maximum fluorescence in each single embryo. Bars in the inset indicate median ± interquartile range.

**(C)** The fluorescence levels of Col IV-GFP in embryos homozygous for the Col IV-GFP trap (Col IV-GFP/Col IV-GFP, magenta) versus those with one copy of Col IV-GFP but heterozygous for the Col IV mutant (ΔColIV) was measured and plotted as in Figure 1C. Note that the reduction of Col IV levels when heterozygous for the ΔColIV mutation shows that there is no compensation for a missing allele. Mean ± SEM. Inset shows quantification of the maximum fluorescence in each single embryo. Bars in the inset indicate median ± interquartile range.

**Table S1. Genotypes of the embryos used in each experiment**

**Related to Figures 1-4, S1-S4, and Movies S1-S4**

| Figure | Panel | Label | Genotype |
|---|---|---|---|
| Figure 1 | A, B | No GFP | $w^{1118}$ |
| | | LanA | LanA-GFP |
| | | Col IV | Col IV-GFP |
| | | Perl | Perl-GFP |
| | C | LanA, Control | LanA-GFP |
| | | LanA, $srp^{AS}$ | LanA-GFP, $srp^{AS}$ |
| | | Col IV, Control | Col IV-GFP |
| | | Col IV, $srp^{AS}$ | Col IV-GFP; $srp^{AS}$ |
| | | Perl, Control | Perl-GFP |
| | | Perl, $srp^{AS}$ | Perl-GFP; $srp^{AS}$ |
| | D, E | | LanA-GFP, Col IV-GFP or Perl-GFP in either control or the homozygous mutant background as indicated |
| Figure 2 | A | LanA | LanA-GFP, sn-Gal4, UAS-CheMoe |
| | | LanB1 | LanB1-GFP, srp-Gal4, UASChe-Moe |
| | | Col IV | Col IV-GFP, srp-Gal4, UAS-Che-Moe |
| | B | LanA | LanA-GFP, sn-Gal4, UAS-CheMoe |
| | | SecrGFP | e22c-Gal4, srp-3xmCherry/+; UAS-secrGFP/+ |
| | | Col IV | Col IV-GFP, srp-Gal4, UAS-CheMoe |
| | | Perl | Perl-GFP; srp-Gal4, UAS-CheMoe |
| | C | SecrGFP | e22c-Gal4, srp-3xmCherry/+; UAS-secrGFP/+ |
| | | LanA | LanA-GFP, srp-3XmCherry/+ |
| | | Col IV | Col IV-GFP |
| | D, E | | $w^{1118}$ |
| | F, G | | Col IV-GFP, srp-3xmCherry |
| Figure 3 | A-C | Control | e22c-Gal4, srp-3xmCherry/+; LanA-GFP/+ |
| | | +Pvf2 | e22c-Gal4, srp-3xmCherry/UAS-Pvf2; LanA-GFP/+ |
| | D-F | Control | e22c-Gal4, Col IV-GFP/Col IV-GFP; srp-3XmCherry/+ |
| | | +Pvf2 | e22c-Gal4, Col IV-GFP/Col IV-GFP, UAS-Pvf2; srp-3XmCherry/+ |
| Figure 4 | A | | $w^{1118}$ |
| | B | Control | $w^{1118}$ |
| | | +Pvf2 | e22c-Gal4/UAS-Pvf2 |
| | C-E | Control | repoCherry/+ |
| | | +Pvf2 | E22cGal4/+; repoCherry/Pvf2 |
| | | RacV12 | repoCherry, sn-Gal4, UAS-LifeAct-GFP/UAS-RacV12 |
| | | RacN17 | repoCherry, sn-Gal4, UAS-LifeAct-GFP/UAS-RacN17 |
| | | ΔCol IV/+ | E22cGal4, ΔCol IV /+; repoCherry/+ |
| | | ΔCol IV/+, +Pvf2 | E22cGal4, ΔCol IV /+; repoCherry/Pvf2 |
| | F | ΔCol IV/+ | ΔCol IV/+ |
| | | RacN17 | srp-Gal4, UAS-Red-Stinger/UAS-RacN17 |
| | | ΔCol IV/+; RacN17 | ΔCol IV/+; sn-Gal4, UAS-Red-Stinger/N17 |
| | | ΔCol IV/ΔCol IV | ΔCol IV/ΔCol IV |
| | | ΔLanB1/+ | ΔLanB1/+ |
| | | ΔLanB1/+; RacN17 | ΔLanB1/+; sn-Gal4, UAS-Red-Stinger/N17 |
| | | ΔLanB1/ΔLanB1 | ΔLanB1/ΔLanB1 |
| Figure S1 | A | NA | NA |
| | B | Control | $lanA^{9-32}$/TM3 |
| | | LanA mut | $lanA^{9-32}/lanA^{MB01129}$ |
| | | LanA mut + LanA-GFP | $lanA^{9-32}/ lanA^{MB01129}$, LanA-GFP |
| | C, D | Control | Col IV-GFP |
| | | ΔLanB1 | Col IV-GFP, ΔLanB1 |
| | E | Col IV | Col IV-GFP |
| | | LanB1 | LanB1-GFP/+ |
| | F | LanA, Control | LanA-GFP |
| | | LanA, $srp^{AS}$ | LanA-GFP, $srp^{AS}$ |
| | | Col IV, Control | Col IV-GFP |
| | | Col IV, $srp^{AS}$ | Col IV-GFP; $srp^{AS}$ |

| | | | |
|---|---|---|---|
| | | Perl, Control | Perl-GFP |
| | | Perl, *srp*$^{AS}$ | Perl-GFP; *srp*$^{AS}$ |
| | G | Control | Col IV-GFP |
| | | ∆LanB1 | Col IV-GFP, ∆LanB1 |
| | H | Control | Col IV-GFP; sn-Gal4, UAS-CheMoe |
| | | ∆LanB1 | Col IV-GFP, ∆LanB1; sn-Gal4, UAS-CheMoe |
| Figure S2 | A | | Col IV-GFP, srp-Gal4, UAS-CheMoe; sn-Gal4, UAS-CheMoe |
| | B | | Col IV-GFP, srp-Gal4, UAS-CheMoe; sn-Gal4, UAS-CheMoe |
| Figure S3 | A | Control | LanA-GFP |
| | | *srp*$^{AS}$ | LanA-GFP, *srp*$^{AS}$ |
| | B | Control | Col IV-GFP/Col IV-GFP, UAS-Pvf2; srp-3XmCherry/+ |
| | | +Pvf2 | e22c-Gal4, Col IV-GFP/Col IV-GFP, UAS-Pvf2; srp-3XmCherry/+ |
| | C, D | Control | Col IV-GFP, srp-Gal4, UAS-CheMoe/Col IV-GFP; sn-Gal4, UAS-CheMoe/+ |
| | | RacV12 | Col IV-GFP, srp-Gal4, UAS-CheMoe/Col IV-GFP; sn-Gal4, UAS-CheMoe/UAS-RacV12 |
| | | RacN17 | Col IV-GFP, srp-Gal4, UAS-CheMoe/Col IV-GFP; sn-Gal4, UAS-CheMoe/UAS-RacN17 |
| | E | Control | Col IV-GFP/Col IV-GFP, UAS-Pvf2; srp-3XmCherry/+ |
| | | +Pvf2 | e22c-Gal4, Col IV-GFP/Col IV-GFP, UAS-Pvf2; srp-3XmCherry/+ |
| Figure S4 | A | Control | srp-Gal4, UAS-Red-Stinger |
| | | Rpr | srp-Gal4, UAS-Red-Stinger X UAS-Rpr |
| | | ZipDN | srp-Gal4, UAS-Red-Stinger/UAS-ZipDN |
| | | RacN17 | srp-Gal4, UAS-Red-Stinger/UAS-RacN17 |
| | | Pvf2 | E22cGal4/UAS-Pvf2;srp-3xmCherry/+ |
| | | RacV12 | srp-Gal4, UAS-Red-Stinger/UAS-RacV12 |
| | B | Control | Col IV-GFP, srp-Gal4, UAS-CheMoe/Col IV-GFP; sn-Gal4, UAS-CheMoe/+ |
| | | RacV12 | Col IV-GFP, srp-Gal4, UAS-CheMoe/Col IV-GFP; sn-Gal4, UAS-CheMoe/UAS-RacV12 |
| | | RacN17 | Col IV-GFP, srp-Gal4, UAS-CheMoe/Col IV-GFP; sn-Gal4, UAS-CheMoe/UAS-RacN17 |
| | C | Col IV-GFP/Col IV-GFP | Col IV-GFP |
| | | Col IV-GFP/∆Col IV | Col IV-GFP/∆Col IV |
| Movie S1 | | No GFP | *w*$^{1118}$ |
| | | Perl | Perl-GFP |
| | | Col IV | Col IV-GFP |
| | | LanA | LanA-GFP |
| Movie S2 | Part 1 | | LanA-GFP, sn-Gal4, UAS-CheMoe |
| | Part 2 | | Col IV-GFP, srp-Gal4, UAS-Che-Moe |
| Movie S3 | Part 1 | | srpGal4, UAS-LifeAct-GFP, srp3XmCherry |
| | Part 2, 3 | | Col IV-GFP, srp-3xmCherry |
| | Part 4 | | Col IV-GFP, srp-Gal4, UAS-CheMoe; sn-Gal4, UAS-CheMoe |
| Movie S4 | Part 1 | LanA | LanA-GFP, srp-3xmCherry |
| | | Col IV | Col IV-GFP, srp-3xmCherry |
| | Part 2 | Control | e22c-Gal4, srp-3xmCherry/+; LanA-GFP/+ |
| | | +Pvf2 | e22c-Gal4, srp-3xmCherry/UAS-Pvf2; LanA-GFP/+ |
| | Part 3 | Control | e22c-Gal4, Col IV-GFP/Col IV-GFP; srp-3XmCherry/+ |
| | | +Pvf2 | e22c-Gal4, Col IV-GFP/Col IV-GFP, UAS-Pvf2; srp-3XmCherry/+ |
| | Part 4 | | e22c-Gal4, Col IV-GFP/Col IV-GFP, UAS-Pvf2; srp-3XmCherry/+ |
| | Part 5 | Control | Col IV-GFP/Col IV-GFP, UAS-Pvf2; srp-3XmCherry/+ |
| | | +Pvf2 | e22c-Gal4, Col IV-GFP/Col IV-GFP, UAS-Pvf2; srp-3XmCherry/+ |
